# Linagliptin-Induced Bullous Pemphigoid: A Case Report and Comprehensive Literature Review

**DOI:** 10.7759/cureus.98011

**Published:** 2025-11-28

**Authors:** Saood Almutairi, Falah M Alajmi, Salman M Albthali, Shehab Aldhafiri

**Affiliations:** 1 Dermatology, Abdulkareem AlSaeed Dermatology Center, Kuwait City, KWT; 2 Dermatology, As'ad Al-Hamad Dermatology Center, Kuwait City, KWT; 3 Dermatology, Al Jahra Hospital, Kuwait City, KWT

**Keywords:** bullous pemphigoid, case report, dupilumab, kuwait, seclusion, targeted therapy

## Abstract

Bullous pemphigoid (BP) is an autoimmune blistering disorder predominantly affecting the elderly, presenting significant management challenges, especially in patients with multimorbidity and polypharmacy. This report details a complex case of a 94-year-old woman with new-onset BP following dipeptidyl peptidase-4 (DPP-4) inhibitor (linagliptin) initiation, complicated by diabetes mellitus, chronic kidney disease, hypertension, and dementia. We describe her clinical course, therapeutic challenges, and response to systemic corticosteroids combined with topical therapies. Furthermore, we comprehensively review the current evidence for emerging biological and targeted therapies (e.g., rituximab, dupilumab, omalizumab, and intravenous immunoglobulin) in BP management, highlighting their potential role in reducing corticosteroid dependence and improving outcomes in frail, comorbid patients. This case underscores the importance of the prompt recognition of drug-induced BP, individualized treatment plans considering comorbidities, and the growing promise of novel therapeutic strategies.

## Introduction

Bullous pemphigoid (BP) is the most common autoimmune blistering disease, with an increasing incidence and a predilection for individuals over 70 years of age [1]. The pathogenesis involves autoantibodies, primarily immunoglobulin G (IgG) and immunoglobulin E (IgE), targeting hemidesmosomal proteins bullous pemphigoid antigen 180 (BP180) and bullous pemphigoid antigen 230 (BP230), leading to complement activation, inflammatory cell recruitment, and subepidermal blister formation [1,2]. Clinically, it presents with severe pruritus and tense bullae on erythematous or normal-appearing skin. The management of BP is notoriously challenging in the elderly due to their frequent comorbidities, such as cardiovascular disease, diabetes, renal impairment, and neurocognitive disorders, which complicate treatment choices [3]. First-line treatment typically involves systemic corticosteroids, but their significant adverse effect profile, including hyperglycemia, osteoporosis, and increased infection risk, drives the search for steroid-sparing and targeted alternatives [4]. Furthermore, an important and well-documented trigger for BP is the use of dipeptidyl peptidase-4 (DPP-4) inhibitors, a common class of diabetic medications, with some cases presenting with a distinct, noninflammatory phenotype [5-10]. This paper presents a challenging case of BP in a nonagenarian with multiple comorbidities, potentially triggered by the DPP-4 inhibitor linagliptin, and reviews the evolving landscape of innovative therapeutic approaches for BP, framing this discussion within the context of managing extremely frail patients where conventional therapy is high risk.

## Case presentation

A 94-year-old woman presented with a widespread, itchy, bullous skin eruption on her limbs and trunk of several weeks' duration. The rash began shortly after initiating the DPP-4 inhibitor linagliptin for diabetes management. Her past medical history was significant for diabetes mellitus, hypertension, chronic kidney disease, hypothyroidism, dementia, gastritis/duodenitis, and status post-knee replacement (2014). Her medication list was extensive and included Caltrate (calcium carbonate), omeprazole, cetirizine, metformin, empagliflozin, amlodipine, levothyroxine, topical fusidic acid, ipratropium nebulizer, codeine/guaifenesin syrup, bisacodyl, intravenous fluids, ceftriaxone, and iron supplements. Notably, linagliptin was a recent addition, and upon suspicion of it being the culprit, it was discontinued, while empagliflozin was continued.

**Figure 1:**
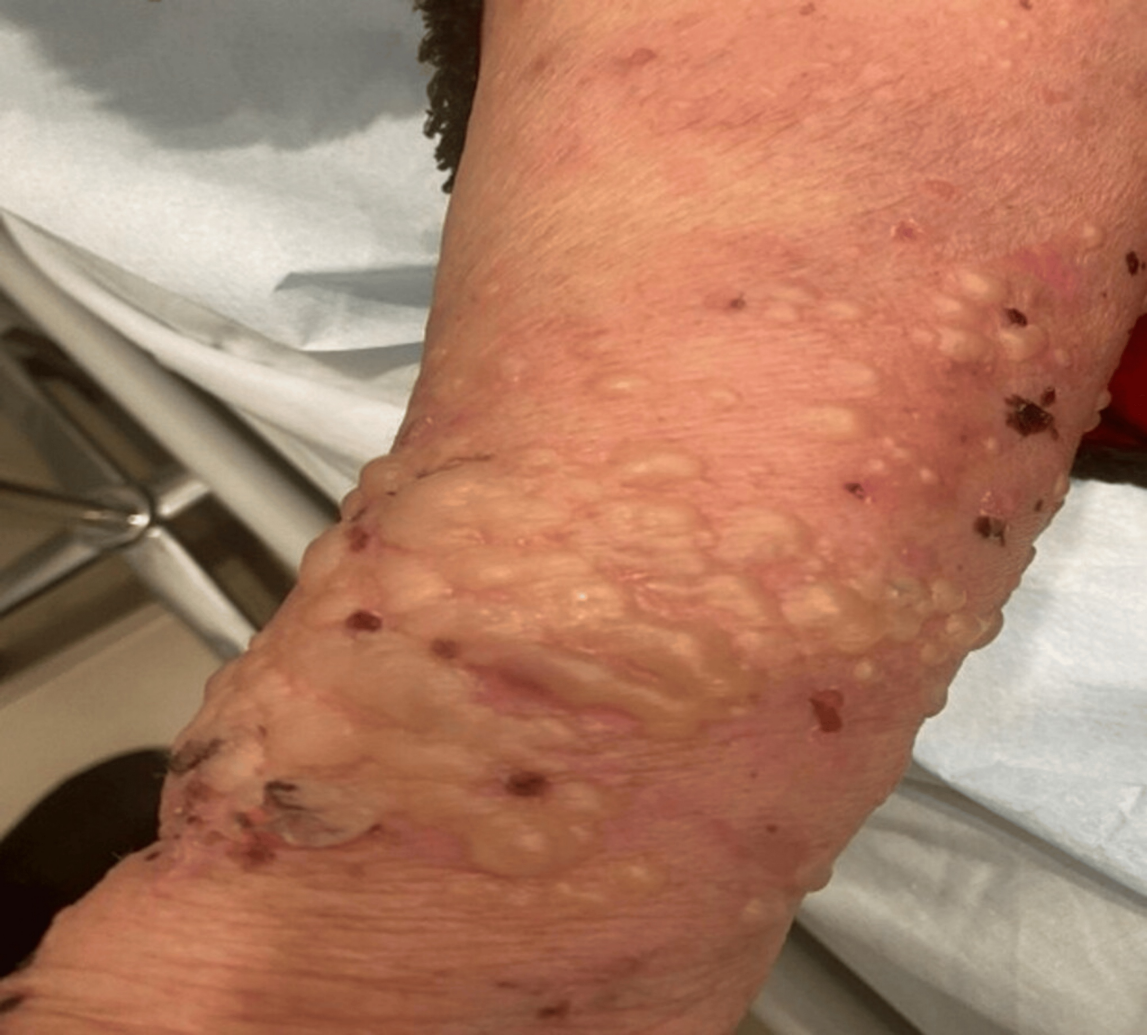
Multiple discrete and closely related tense bullae on normal and erythematous skin with crusted erosions due to ruptured bullae on the forearms and wrist.

Figure 1 demonstrates multiple discrete, tense bullae on both erythematous and normal skin, accompanied by crusted erosions from ruptured lesions on the forearms and wrists. These cutaneous findings are consistent with the classic clinical presentation of BP, characterized by widespread tense blistering and intense pruritus. Mucosal surfaces were spared, and Nikolsky's sign was negative.

**Figure 2:**
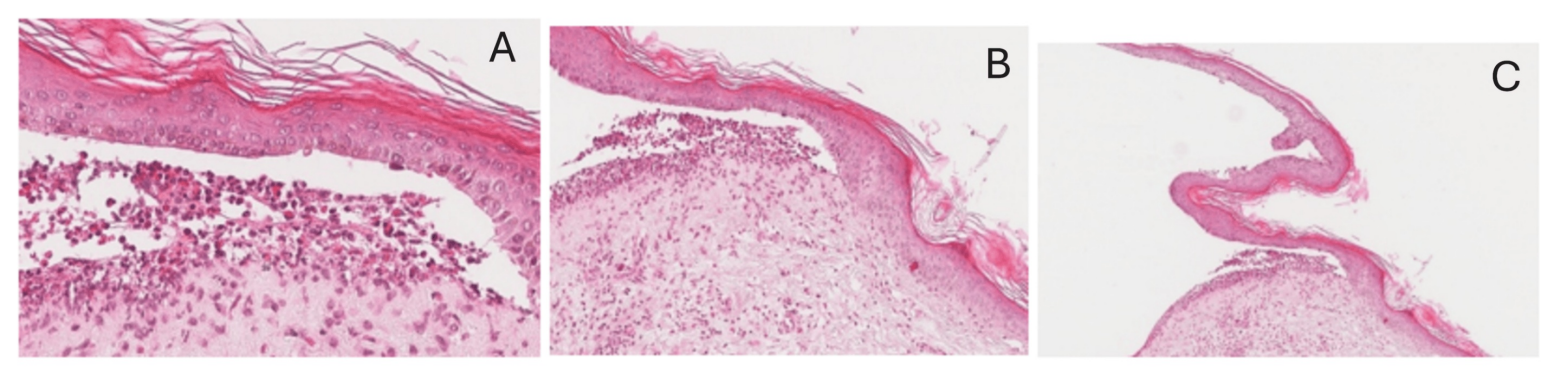
Histopathology (hematoxylin and eosin {H&E} stain) demonstrates the subepidermal clefting, resulting in a tense blister cavity. Within the blister space, eosinophils and lymphocytes are prominently observed (A and B).

Histopathological analysis, shown in Figure 2A-2C, revealed a subepidermal blister cavity with abundant eosinophils and lymphocytes, confirming the inflammatory nature of the disease process.

**Figure 3:**
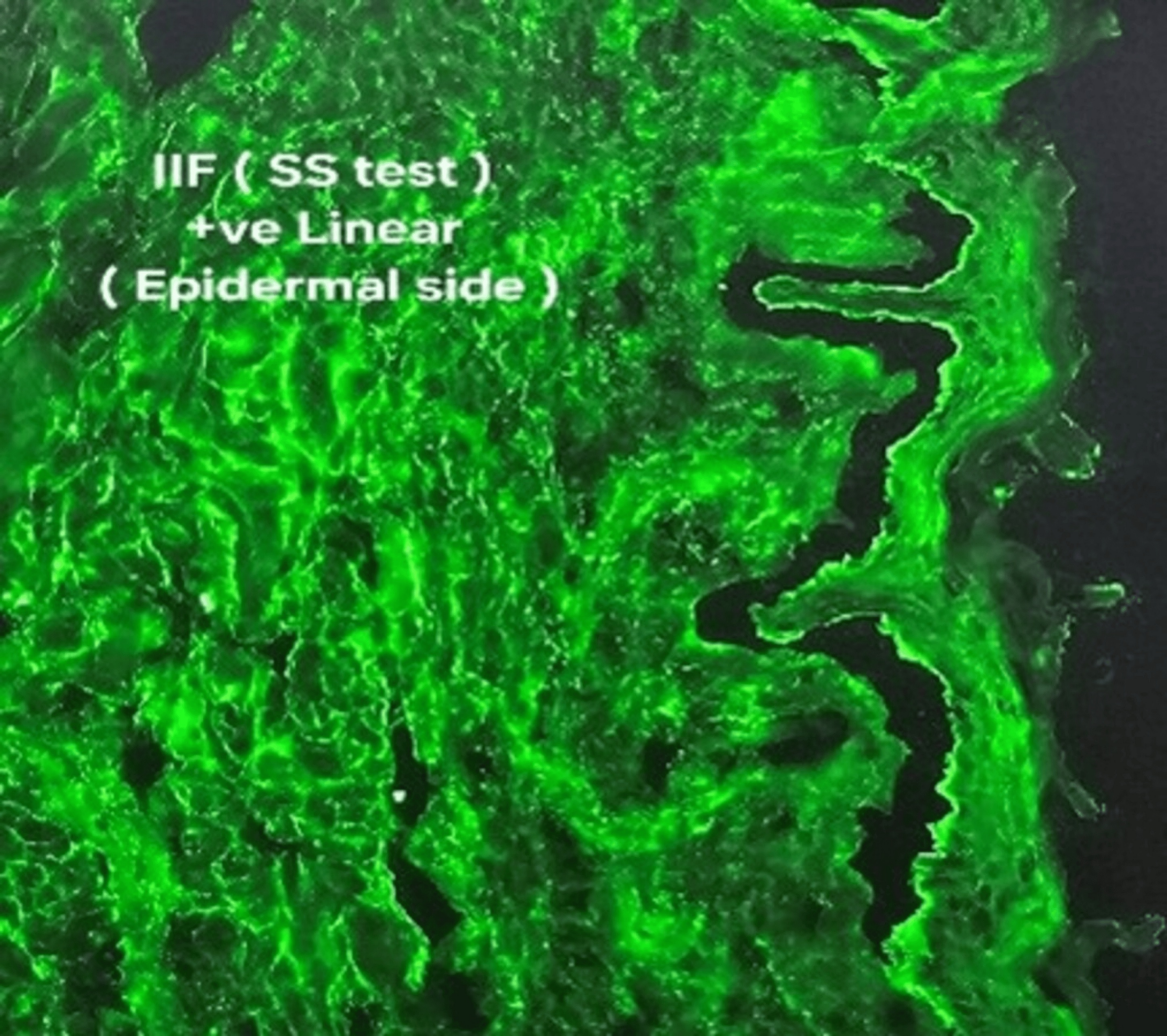
Indirect immunofluorescence (IIF) on both monkey esophagus (MO) and salt-split skin (SS) substrates confirms circulating autoantibodies with epidermal-side binding. These results collectively support a diagnosis of bullous pemphigoid.

Indirect immunofluorescence (IIF) results, illustrated in Figure 3, on monkey esophagus and salt-split skin substrates confirmed circulating autoantibodies with epidermal-side binding, findings diagnostic of BP.

Initial management included oral prednisolone (30 mg daily), potent topical corticosteroids (clobetasol propionate), and topical antibiotics (fusidic acid). The patient showed gradual improvement; the bullae crusted and healed, with no new lesions forming after one week. Oral prednisolone was tapered over four weeks, and the patient was maintained on topical corticosteroids until complete clearance. The Naranjo Adverse Drug Reaction Probability Scale score was 6, indicating a "probable" association between linagliptin and BP. Supportive care for anemia, hypomagnesemia (magnesium sulfate), constipation, and deep vein thrombosis (DVT) prophylaxis (enoxaparin) was crucial. Incidentally noted hematuria and left eye ptosis were investigated and deemed unrelated to BP or its treatment.

## Discussion

The presented case of a 94-year-old woman with BP illustrates the profound complexities of managing this disease in the context of advanced age, multimorbidity, and polypharmacy. The temporal association between linagliptin initiation and rash onset, coupled with a Naranjo score of 6, is highly suggestive of DPP-4 inhibitor-induced BP, warranting its immediate discontinuation [5,11].

Management and therapeutic rationale

Our management strategy prioritized minimizing systemic steroid exposure. While guidelines often recommend initial doses of 0.5-1 mg/kg of prednisolone, we initiated a lower dose of 30 mg (approximately 0.3-0.4 mg/kg) due to the patient's age, chronic kidney disease, and dementia. This was effective in achieving disease control, demonstrating that a tailored, lower-dose regimen can be successful in very frail patients. Topical clobetasol propionate and fusidic acid provided a crucial adjunctive benefit. This case underscores the limitations and risks of conventional therapy, even at lower doses, highlighting the need for steroid-sparing strategies.

Pathogenesis of drug-induced bullous pemphigoid

The mechanism by which DPP-4 inhibitors trigger BP is not fully elucidated but is thought to involve molecular mimicry. The DPP-4 enzyme shares structural homology with BP180, potentially leading to the generation of cross-reactive autoantibodies. Furthermore, DPP-4 inhibition may alter immune regulation and the inflammatory microenvironment, lowering the threshold for blister formation in predisposed individuals [5,12,13].

Review of emerging therapeutic strategies

For frail, elderly patients with contraindications to conventional immunosuppressants, newer biologics offer significant promise. Table 1 provides a comparative overview of these therapies.

**Table 1:** Emerging therapies for bullous pemphigoid. CD20, cluster of differentiation 20; IV, intravenous; IL-4Rα, interleukin 4 receptor alpha; IgE, immunoglobulin E; IVIG, intravenous immunoglobulin

Therapy	Mechanism of Action	Key Efficacy Findings	Safety Considerations	Cost and Accessibility
Rituximab	Anti-CD20 monoclonal antibody; depletes B-cells	Highly effective in refractory cases; promotes long-term remission	Increased risk of infections; requires premedication	High cost; requires IV infusion
Dupilumab	Anti-IL-4Rα monoclonal antibody; blocks IL-4/IL-13 signaling	Rapid pruritus control; effective blister control; strong steroid-sparing effect	Favorable safety profile; conjunctivitis is a known side effect	High cost; subcutaneous administration
Omalizumab	Anti-IgE monoclonal antibody	Effective in case series, especially with high IgE/eosinophilia	Well-tolerated; anaphylaxis risk is rare	High cost; subcutaneous administration
IVIG	Polyvalent IgG; multiple immunomodulatory effects	Effective as a steroid-sparing agent in refractory disease	Good safety profile (non-immunosuppressive); risk of infusion reactions	Very high cost; requires IV infusion; limited supply

Rituximab

While approved for pemphigus vulgaris, its use in BP is supported by growing evidence and guideline recommendations for refractory cases. Its use in nonagenarians requires careful infection risk assessment.

Dupilumab

By targeting the interleukin 4 (IL-4)/IL-13 pathway, it rapidly controls pruritus and blistering. Its favorable safety profile makes it a promising first-line biologic for BP.

Omalizumab

This anti-IgE antibody is particularly useful in patients with elevated IgE levels and offers another steroid-sparing option.

*Other Agents (Bruton's Tyrosine Kinase* {*BTK} Inhibitors and Neonatal Fragment Crystallizable Receptor {FcRn} Antagonists)*

These represent the next frontier of targeted therapy but remain in early phases of investigation for BP.

## Conclusions

Managing bullous pemphigoid in elderly, multimorbid patients requires a highly individualized approach balancing efficacy and safety. The presented case highlights that a lower-dose corticosteroid regimen, combined with topical therapy and the removal of the offending drug, can be effective even in a nonagenarian. DPP-4 inhibitors must be recognized as potential triggers. The advent of targeted biological therapies offers transformative potential for improving outcomes in more severe or refractory cases by providing potent, steroid-sparing alternatives. Future management strategies should consider these innovative agents to minimize steroid side effects in complex patients while considering factors such as cost and accessibility.
